# Magnetic Resonance Imaging of the Ear for Patient-Specific Reconstructive Surgery

**DOI:** 10.1371/journal.pone.0104975

**Published:** 2014-08-21

**Authors:** Luc Nimeskern, Eva-Maria Feldmann, Willy Kuo, Silke Schwarz, Eva Goldberg-Bockhorn, Susanne Dürr, Ralph Müller, Nicole Rotter, Kathryn S. Stok

**Affiliations:** 1 Institute for Biomechanics, ETH Zurich, Zurich, Switzerland; 2 Department of Otorhinolaryngology, Ulm University Medical Center, Ulm, Germany; 3 Department of Diagnostic and Interventional Radiology, Ulm University Medical Center, Ulm, Germany; University of Pécs Medical School, Hungary

## Abstract

**Introduction:**

Like a fingerprint, ear shape is a unique personal feature that should be reconstructed with a high fidelity during reconstructive surgery. Ear cartilage tissue engineering (TE) advantageously offers the possibility to use novel 3D manufacturing techniques to reconstruct the ear, thus allowing for a detailed auricular shape. However it also requires detailed patient-specific images of the 3D cartilage structures of the patient’s intact contralateral ear (if available). Therefore the aim of this study was to develop and evaluate an imaging strategy for acquiring patient-specific ear cartilage shape, with sufficient precision and accuracy for use in a clinical setting.

**Methods and Materials:**

Magnetic resonance imaging (MRI) was performed on 14 volunteer and six cadaveric auricles and manually segmented. Reproducibility of cartilage volume (Cg.V), surface (Cg.S) and thickness (Cg.Th) was assessed, to determine whether raters could repeatedly define the same volume of interest. Additionally, six cadaveric auricles were harvested, scanned and segmented using the same procedure, then dissected and scanned using high resolution micro-CT. Correlation between MR and micro-CT measurements was assessed to determine accuracy.

**Results:**

Good inter- and intra-rater reproducibility was observed (precision errors <4% for Cg.S and <9% for Cg.V and Cg.Th). Intraclass correlations were good for Cg.V and Cg.S (>0.82), but low for Cg.Th (<0.23) due to similar average Cg.Th between patients. However Pearson’s coefficients showed that the ability to detect local cartilage shape variations is unaffected. Good correlation between clinical MRI and micro-CT (r>0.95) demonstrated high accuracy.

**Discussion and Conclusion:**

This study demonstrated that precision and accuracy of the proposed method was high enough to detect patient-specific variation in ear cartilage geometry. The present study provides a clinical strategy to access the necessary information required for the production of 3D ear scaffolds for TE purposes, including detailed patient-specific shape. Furthermore, the protocol is applicable in daily clinical practice with existing infrastructure.

## Introduction

A key aspect of tissue-engineering (TE) strategies and reconstructive surgery of the ear, nose and throat (ENT) is the final shape of the reconstructed organ. Since aesthetics and patient satisfaction are critical criteria of success for these procedures [1], [2], patient-specific organ shape alongside long-term shape stability must be achieved. Like a fingerprint, ear shape is a unique personal feature [3]–[5] that should be reconstructed with a high fidelity. This has been recognized as a particularly acute issue for the outer ear [6]–[9] due to its “*complex architecture and largely unsupported, protruding, three-dimensional structure*” [9].

To repair trauma affecting the outer part of the ear for patients with high comorbidity, epitheses are typically used [10], [11] to minimize the risks of complications. Otherwise, surgical solutions are the only other alternatives available today. For this, there are two approaches – autologous reconstruction and synthetic implants [12]. In autologous reconstruction, autologous cartilage is used to create a 3D cartilage framework that mimics the intact contralateral ear which is then implanted subcutaneously at the defect site [10], [12]–[15]. However this procedure presents a significant complication risk, in particular for total ear reconstruction due to donor site morbidity following cartilage harvesting [16]. Additionally the aesthetic outcome is highly dependent on the skill of the surgeon, because the implanted ear framework is made of assembled autologous cartilage pieces carved by hand [17]. Alternatively synthetic implants such as polyethylene implants [18] can be implanted with a better cosmetic outcome but higher risk for infection and extrusion [19], [20]. TE applied to reconstructive surgery has gained recently wide attention as a way to alleviate these shortcomings [21]. Ear cartilage TE would potentially obviate the need for hand-carved cartilage frameworks or synthetic implants, by replacing these with a cell-seeded artificial scaffold [21]. Various strategies have been investigated, such as polymeric scaffolds [7], hydrogels [8] or biodegradable scaffolds with a non-biodegradable core [9]. TE advantageously offers also the possibility to use manufacturing techniques for scaffold production [6], which allow more detailed and controlled shapes. However, as such scaffolds are meant to be implanted subcutaneously in order to replace the lost cartilage, these should not be made in the shape of the patient external ear but in the shape of its internal cartilage structure. Hence for an optimal outcome, TE scaffold manufacturing should be combined with 3D imaging techniques so as to obtain detailed and patient-specific scaffolds that mimic the cartilage structure of the patient’s intact contralateral ear (if available).

Protocols are already available for the production of 3D scaffolds with customizable shape using computer-aided design and manufacturing techniques [6], [22]. For example, Reiffler et al. [22] demonstrated the production of collagen type I scaffolds with patient-specific ear shape. However, imaging techniques such as computed tomography [6] (CT) or digital photogrammetry [22] are limited to the external ear shape. The use of MRI for rapid prototyping of 3D ear epitheses was also reported, although here as well the authors aim at reproducing the external ear shape and not its specific tissue structures [23], [24], i.e. skin, fat and cartilage tissues were imaged as one structure, and no information about the unique cartilage structure present in the auricle was obtained. [23], [24]Therefore, in this study, the aim is to develop an imaging protocol that allows for segmentation of ear cartilage only and uses resources that are clinically available. Magnetic resonance imaging (MRI), which is a state of the art non-invasive modality for articular cartilage diagnostics, has been identified as a promising technique due to its good soft tissue contrast and widespread availability [25]–[27].

In order to characterize the quality of an imaging strategy it is necessary to assess its precision and accuracy [28]. Precision is a measure of the error introduced by the operators performing the measurement and analysis. This is important if segmentation of cartilage, whether manual or computer-based, is required. In other words, measures of precision assess whether the 3D ear cartilage shape obtained depends on the operator involved [29]. Accuracy evaluation compares the new method to a standard for high-resolution 3D imaging, such as micro-computed tomography [30] (micro-CT). This indicates how close the 3D ear cartilage shape, obtained with the new strategy, is to true cartilage shape.

The present study aims to identify a potential clinical solution for patient-specific ear shape imaging and to evaluate whether this new strategy can be applied with sufficient accuracy and precision in a clinical setting. It will be assessed whether switching the personnel dedicated to this task (the raters) affects the evaluation of the ear cartilage shape (inter- and intra-rater precision) and whether this method characterizes the true shape of the ear cartilage (accuracy using micro-CT as the standard).

## Materials and Methods

### Ethics Statement

All subjects gave their written informed consent to the study. The study was approved by the institutional review board of the Ulm University (Ethikantrag 150/12, Ethical Committee, Ulm University).

### Identification of an imaging strategy of human ear cartilage

Pilot work was conducted in order to identify the optimal MRI sequences for imaging of human ear cartilage. This sequence must provide resolution and contrast high enough to visually distinguish ear cartilage from the surrounding tissues (perichondrium, skin and adipose tissue). MRI scans of the ear were performed on healthy volunteers with a clinical MRI (Magnetom Skyra 3T, Siemens AG, Erlangen, Germany) equipped with a head coil (Head/Neck 20, A 3T Tim Coil). Four different sequences were acquired (n = 1, each); spoiled gradient-echo with (FS-SGE) and without (SGE) fat saturation, SPACE (Sampling Perfection with Application optimized Contrasts by using different flip angle Evolutions) and MPRAGE (Magnetization Prepared Rapid Acquisition Gradient Echo). Additionally, FS-SGE sequences at 0.45 mm and 0.30 mm resolution were acquired (n = 3, each). The best visualization of ear cartilage was obtained with the FS-SGE sequences. Comparison between 0.30 mm and 0.45 mm resolution showed that at 0.30 mm resolution, the lower signal to noise ratio limited the ability to visualize ear cartilage despite a smaller voxel size. None of the pilot scans displayed sufficient contrast and resolution to allow for automated computer-based segmentation. Therefore, clinical MRI imaging with a FS-SGE sequence combined with manual segmentation was selected as a potential clinical solution for patient-specific ear shape imaging.

### Precision measurement

MRI scanning was performed on the left ear of 14 volunteers with a clinical MRI system (Magnetom Skyra 3T, Siemens AG, Erlangen, Germany) equipped with a head coil (Head/Neck 20, A 3T Tim Coil). All volunteers gave written informed consent for the study. A FS-SGE sequence was used with an in-plane (XY, sagittal plane) resolution of 0.45 mm x 0.45 mm and an out-of-plane (Z, orthogonal to the sagittal plane) resolution of 0.40 mm.

Datasets were scaled up five times in the X and Z directions in order to allow more precise manual contouring in a later step. For each scan, the operators performing manual segmentation were asked to browse the image stack and manually delineate the ear cartilage which would provide a mask of the tissue (micro-CT Evaluation Program 6.5–1 for VMS; Scanco Medical AG, Brüttisellen, Switzerland). Each operator was first trained on three datasets, which were not included in the subsequent analysis. Three different raters (referred to as rater 1, rater 2 and rater 3) independently segmented all 14 scans once (total of 42 masks). Additionally, rater 1 segmented all 14 scans three times. This yielded in one hand, 3 sets of 14 masks created by 3 different raters, which were used to assess whether masks obtained by the different raters were similar (inter-rater reproducibility). On the other hand, the 3 sets of 14 masks created by the same rater (rater 1) were used to assess whether one rater was able to reproduce the same result multiple times (intra-rater reproducibility).

In order to compare the masks obtained by the different raters, morphological characterization was performed. The masks were scaled isotropically, and cartilage volume (Cg.V), surface (Cg.S), and mean thickness (Cg.Th) were computed using in-house scripts, as described previously [28]. Photographs of the left ears of all 14 volunteers were taken for visual comparison with the segmented datasets.

### Accuracy measurement

Six cadaveric auricles were harvested by Science Care (Phoenix, Arizona, USA, n = 4) and the Erasmus Medical Center (Rotterdam, The Netherlands, n = 2). In line with the ethical guidelines of the respective institutions (Ethikantrag 150/12, Ethical Committee, Ulm University), harvesting of human material was performed with prior written informed consent of the donor.

The 6 cadaveric auricles were immersed in an agarose gel, in order to enhance the signal to noise ratio, and scanned using the clinical MRI procedure described above. Manual segmentation was performed by rater 1, and Cg.V, Cg.S, and Cg.Th were computed as described above.

After scanning, the auricles were dissected in order to remove all tissue surrounding the cartilage and immersed overnight in a solution of PBS with 40% Hexabrix (Mallinckrodt Inc., St Louis, MO, USA). Hexabrix is a clinical contrast agent used routinely in MRI. In the present setup, Hexabrix increases the X-ray absorption of the cartilage, resulting in improved contrast in the subsequent micro-CT scan. One percent antimycotic-antibiotic (Gibco, Life Technologies, Carlsbad, CA, USA) was added to the solution to prevent tissue degradation. The dissected ear cartilage was scanned in air using micro-CT (µCT100, Scanco Medical AG, Brüttisellen, Switzerland) with an isotropic voxel size of 36.8 µm, 45 kVp energy, 88 µA intensity and 200 ms integration time. Threefold frame averaging was selected to improve the signal-to-noise ratio. A Gaussian filter (σ = 1.2, support = 1) and a threshold (linear attenuation between 0.9 cm^−1^ and 2.2 cm^−1^) were then applied to segment the dissected cartilage from the background noise. Cg.V, Cg.S, and Cg.Th were computed as described above.

### Statistical analysis

Precision error (PE) calculation in imaging technology was introduced by Glüer et al. to characterize the reproducibility of a given measurement technique [29]. For inter-rater and intra-rater precision studies, PE was calculated for Cg.V, Cg.S, and Cg.Th and expressed as a percentage of the coefficient of variation of the repeated measurements [31] (PE_%CV_). A set of 14 measurements with three raters ensures 27 degrees of freedom as recommended [29]. The intraclass correlation coefficient (ICC, ranging from 0 to 1) was computed using a two-way model (absolute agreement for single measurements) as described previously [32]. For both inter-rater and intra-rater reproducibility, PEs describe the variations between the three masks obtained for any patient, and ICCs whether the precision is high enough to detect differences between patients. Additionally Pearson’s correlation coefficient (r) was used for pairwise comparisons of the masks obtained in both reproducibility studies [33]. Pearson’s correlation coefficient indicates whether raters consistently identify local variations in the contoured shape.

All statistics were performed with the SPSS software package (SPSS 20.0 for Windows; SPSS Inc., Chicago, IL, USA). All results are displayed as mean ± standard deviation.

## Results

### Identification of an imaging strategy of human ear cartilage

In a preliminary study a potential clinical solution for patient-specific ear shape imaging was identified. This solution combines clinical MRI (FS-SGE sequence) with an in-plane (XY, sagittal plane) resolution of 0.45 mm x 0.45 mm and an out-of-plane (Z, orthogonal to the sagittal plane) resolution of 0.40 mm, with manual segmentation to acquire the ear cartilage image.

### Precision measurement

**Figure 1:**
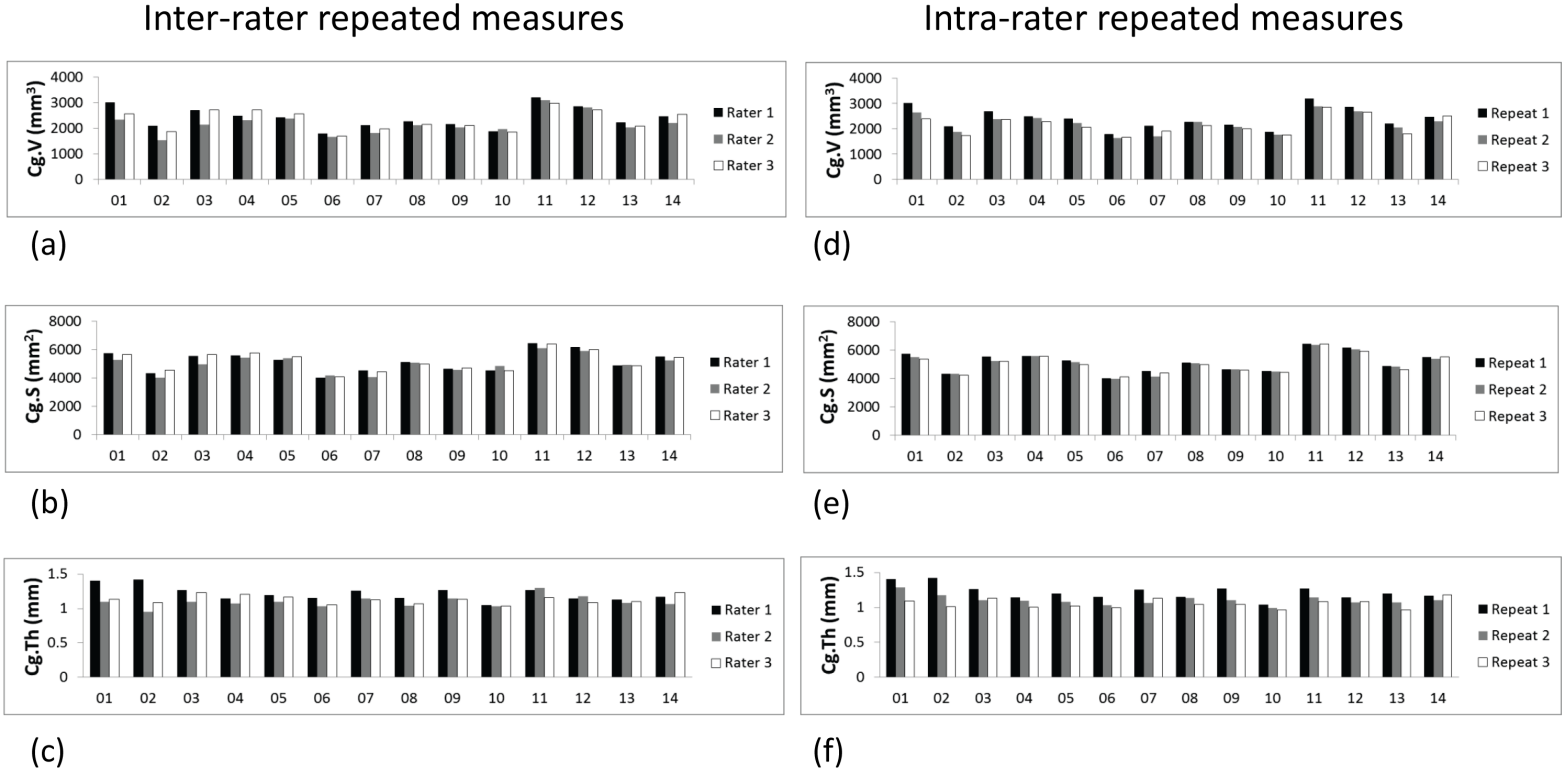
Inter-rater (a, b c) and intra-rater (d, e, f) repeated measures for cartilage volume (Cg.V), cartilage surface (Cg.S) and mean cartilage thickness (Cg.Th) for all 14 volunteers. Good reproducibility is observed for Cg.V and Cg.S values. Additionally low patient-to-patient variations are observed for Cg.Th, i.e. Cg.Th for all 14 volunteers and all three raters is 1.15±0.10 mm, whereas the average Cg.V and Cg.S are 2295±415 mm^3^ and 5102±667 mm^2^, respectively.

Each operator demonstrated a steep learning curve while working on the three training datasets, being able to identify cartilage after the third training dataset. Cg.V, Cg.S, and Cg.Th measured on all 14 volunteers are displayed in Figure 1. Figure 1 a, b, c shows values obtained by the three different raters (inter-rater reproducibility), while Figure 1 d, e, f displays the three repeated measurements performed by rater 1 (intra-rater reproducibility). High similarity between the three repeated measurements obtained for all patients were observed. Additionally, average Cg.V and Cg.S are 2295±415 mm^3^ and 5102±667 mm^2^ for all 14 volunteers and all three raters, respectively, while, small patient-to-patient variations (i.e. low standard deviation) were observed for cartilage thickness, i.e. average Cg.Th was 1.15±0.10 mm.

Corresponding PE_%CV_ and ICC values are displayed in Tables 1 and 2. For both the inter-rater and the intra-rater reproducibility PE_%CV_ were below 4% for Cg.S and below 9% for Cg.V and Cg.Th. Higher precision in Cg.V and Cg.S was observed for intra-rater when compared to inter-rater reproducibility (e.g. inter-rater vs. intra-rater PE_%CV_ is 7.98% vs. 7.19% for Cg.V, and 3.74% vs. 2.33% for Cg.S). ICC values obtained in both inter-rater and intra-rater measurements were satisfying for Cg.V (0.82 and 0.84 respectively), very good for Cg.S (0.92 and 0.97 respectively), but very low for Cg.Th (0.08 and 0.23 respectively).

**Table 1:** Mean, precision error (PE_SD_), precision error expressed as a percentage of coefficient of variation of the repeated measurements (PE_%CV_) and intraclass correlation coefficient (ICC) computed using a two-way model (absolute agreement for single measurements) for inter-rater reproducibility.

Inter-rater reproducibility	Cg.V	Cg.S	Cg.Th
**Mean**	2295.3 mm^3^	5101.8 mm^2^	1.15 mm
**PE_SD_**	182.6 mm^3^	188.6 mm^2^	0.10 mm
**PE_%CV_**	7.98%	3.74%	8.45%
**ICC**	0.82	0.92	0.08

**Table 2:** Mean, PE_SD_, PE_%CV_ and ICC for intra-rater reproducibility.

Intra-rater reproducibility	Cg.V	Cg.S	Cg.Th
**Mean**	2253.7 mm^3^	5073.4 mm^2^	1.13 mm
**PE_SD_**	163.6 mm^3^	116.2 mm^2^	0.10 mm
**PE_%CV_**	7.19%	2.33%	8.87%
**ICC**	0.84	0.97	0.23

PE_%CV_ values are below 5% for Cg.S and below 10% for Cg.V and Cg.Th which demonstrates good precision. ICC values obtained in both inter-rater and intra-rater measurements are good for Cg.V and Cg.S, but very low for Cg.Th. This indicates that the proposed method is adequate to distinguish patient-specific variations for Cg.V and Cg.S only.

**Figure 2:**
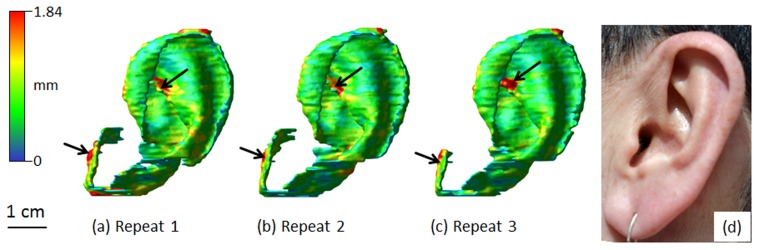
(a,b,c) Thickness maps of a typical set of three ear cartilage masks obtained by clinical MRI imaging combined with manual segmentation, all 3 masks were obtained by rater 1 (intra-rater repeated measures). (d) Corresponding photograph of the volunteer ear. Identical shapes (a, b, c and d) as well as matching regions of higher thickness (a, b and c) can be observed (arrows). Scale bar: 1 cm.

Figure 2 (a, b and c) shows thickness maps of a set of three repeated ear cartilage masks obtained by rater 1 for a typical volunteer, additionally Figure 2 (d) displays a photograph of the corresponding volunteer ear. Identical gross shape (a, b, c and d) as well as matching regions of thickness variation (a, b and c) can be observed.

**Figure 3:**
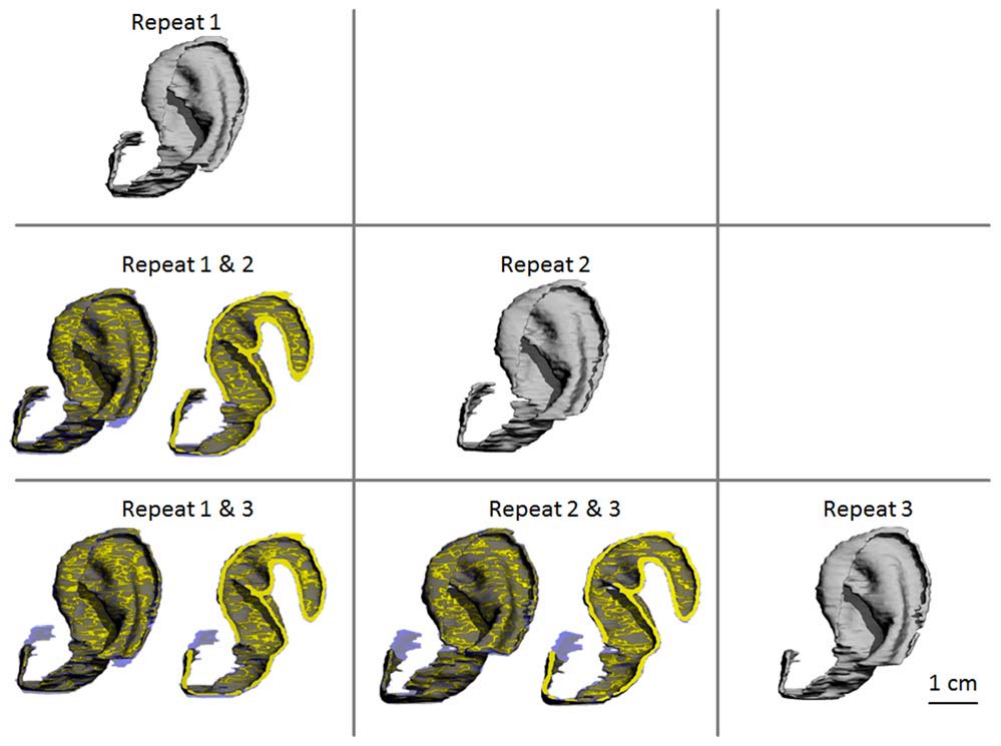
Pairwise comparisons of the three repeated masks obtained by rater 1 for a typical volunteer. The three masks obtained by segmentation are display in the diagonal. These masks are superimposed two-by-two, each overlapped pair of masks (left-hand side) and a corresponding cross-section (right-hand side) are displayed. Common regions are represented in yellow. The areas that belong to only one of the two masks are semi-transparent. Large common regions are observed in all three overlapped pairs of masks, i.e. the overall integrity of shape is maintained, with minor surface variations. This demonstrates that the inability to detect patient-specific variation for mean Cg.Th (low ICC) does not adversely affect the capacity of the proposed clinical method to detect local shape variations within a volunteer auricle. Scale bar: 1 cm.

Good Pearson’s coefficients for pairwise comparison within inter-rater reproducibility study (average r value for 42 pairwise combinations: 0.75±0.03) and the intra-rater (average r value for 42 pairwise combinations: 0.81±0.03) were obtained. Figure 3 displays the pairwise comparisons of the three repeated masks obtained by rater 1 for a typical volunteer, where it can be seen that the overall integrity of shape was maintained, with minor surface variations.

### Accuracy measurement

**Figure 4:**
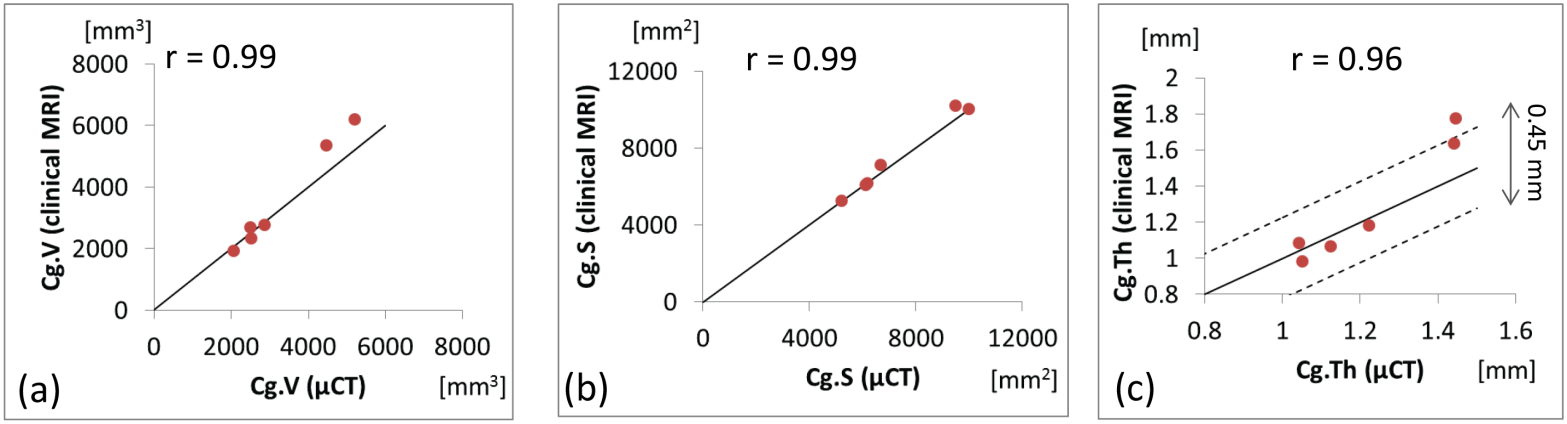
Correlation between values measured with contrast-enhanced micro-CT (after dissection) and values obtained by clinical MRI imaging combined with manual segmentation. Solid line represents y = x. (a) A correlation of r = 0.99 is observed for cartilage surface (Cg.S), (b) r = 0.99 for cartilage volume (Cg.V), (c) r = 0.96 for mean cartilage thickness (Cg.Th), dotted lines represented a 1-voxel error (0.45 mm) on the MRI datasets.

**Figure 5:**
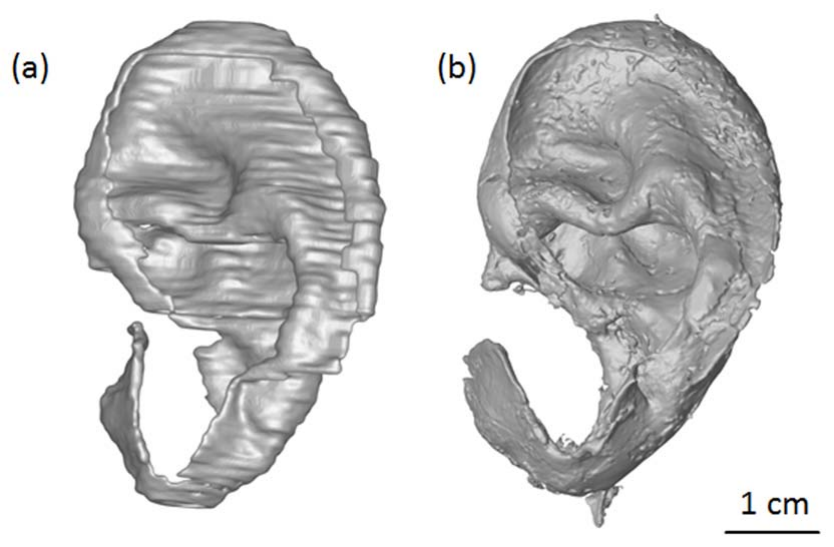
Visual comparison between ear cartilage masks used for accuracy measurement. (a) Cartilage mask obtained by clinical MRI imaging combined with manual segmentation, (b) mask obtained on the same cadaveric sample with contrast-enhanced micro-CT (after ear dissection). Scale bar: 1 cm.

Figure 4 displays the values of Cg.V, Cg.S, and Cg.Th obtained by segmentation of the MRI datasets against the corresponding values obtained with micro-CT. Good correlation was observed between clinical MRI and micro-CT measurements of Cg.V (r = 0.99), Cg.S (r = 0.99), and Cg.Th (r = 0.96), see figure 4 and 5.

## Discussion

The PE_%CV_ values (≤10%) observed for both inter- and intra-rater reproducibility demonstrated good precision (see Tables 1 and 2), i.e. the raters were able to repeatedly define the same volume of interest. Higher precision in Cg.V and Cg.S was observed for intra-rater when compared to inter-rater reproducibility (see Tables 1 and 2) which indicates that variations were smaller when only one rater was involved in the segmentation task. Additionally, whether inter- or intra-rater reproducibility measurements were considered, precision for Cg.S was the highest, followed by Cg.V and finally Cg.Th. To explain this ranking, two observations are necessary. Firstly, the limited spatial resolution of the MRI datasets (0.45 mm), compared to the measured mean cartilage thickness (1.13±0.11 mm), explains the low precision of the mean thickness measurements. Secondly, as ear cartilage shape can - in a first approximation - be considered as a layer-like structure (i.e. a curved sheet of cartilage with homogeneous thickness), Cg.V can be approximated as the product of Cg.S by Cg.Th. This implies that the measurement error on Cg.Th will propagate to Cg.V leading to a lower precision for Cg.V than for Cg.S.

From the measured PE_%CV_ it can be concluded that using the proposed technique all three parameters of interest can be measured with satisfying precision. As the gain in precision observed between intra- and inter-rater measurements is limited (see Tables 1 and 2), in a clinical set-up different personnel could be involved in this task without adversely influencing the outcome. Nevertheless, initial training will be very important to reduce error and increase consistency.

Similarly, better ICC was obtained for intra-rater repeated measures than for inter-rater repeated measures, which is a direct consequence of the better PE_%CV_ observed for intra-rater measurements. ICC values obtained in both inter- and intra-rater measurements were good for Cg.V (≥0.8) and for Cg.S (≥0.9), but very low for Cg.Th (≤0.3). ICC characterizes whether the precision of a measurement (i.e. its PE_%CV_ value) is good enough to detect variations between patients. Therefore the proposed clinical method is adequate to distinguish patient specific variations for Cg.V and Cg.S. However the precision of Cg.Th is too low for the detection of variations in mean cartilage thickness between patients. This is a combined effect of the low precision of Cg.Th (due to low MRI resolution, as explained earlier) and of the small patient to patient variation in mean cartilage thickness, see Figure 1 (a). However, despite low ICC for Cg.Th, satisfying pairwise correlations were observed for both inter- and intra-rater measurements, see Figure 3. This indicates that the inability to detect patient-specific variation of mean cartilage thickness does not affect the capacity of the proposed clinical method to detect local shape variations within an auricle.

In order to assess whether the proposed method for ear shape imaging is applicable in a clinical procedure for ear TE, its accuracy must also be evaluated. In other words, the masks produced with this MRI protocol have to be compared with the actual ear cartilage shape. There are no standard methods for quantitative imaging of ear cartilage. In literature the use of techniques such as clinical CT [6], digital photogrammetry [22] or MRI was limited to the acquisition of external ear shape only [23], [24], As opposed to MRI, high resolution micro-CT imaging provides high spatial resolution down to the micrometer range [34], but on the other hand, soft-tissue contrast is low when compared to MRI. The use of contrast agents such as Hexabrix makes micro-CT imaging of soft-tissues possible [35]. Thanks to its greater spatial resolution, contrast enhanced high resolution micro-CT combined with dissection was used as a standard to assess the accuracy of the method. After dissection only the cartilage remains, therefore there is no need to segment it. Using contrast-enhanced micro-CT, cartilage is then readily imaged with a resolution more than ten times higher than MRI (36.8 µm for micro-CT vs. 450 µm for MRI), see figure 5. The good correlation observed between clinical MRI and micro-CT measurements of Cg.V, Cg.S, and Cg.Th show that the values obtained by the proposed clinical method can accurately predict ear cartilage thickness, surface and volume. As seen in Figure 4, Cg.Th values measured with the proposed clinical method differ by less than 0.4 mm (the spatial resolution of the MRI datasets) from their micro-CT counter parts. These values are very satisfying considering the resolution limitation inherent to clinical MRI. These results show that the new imaging strategy proposed is able to characterize the patient-specific ear cartilage shape.

In conclusion, clinical MRI imaging combined with manual segmentation of ear cartilage was demonstrated to be accurate. The precision of this new strategy was high enough to detect patient-specific variation in ear cartilage surface and volume, as well as local shape variations within a volunteer auricle. Precision was additionally shown to be independent of the personnel dedicated to the manual segmentation. Therefore, in a clinical set-up, different personnel could be involved in this task without adversely influencing the outcome. Finally, as the only requirements for this strategy are the access to clinical MRI and personnel for ear cartilage segmentation, this method is applicable in daily clinical practice with existing infrastructures. Alongside novel TE strategies currently under development [7]–[9], the resulting 3D ear masks have the potential to improve aesthetic outcomes of surgical reconstruction; and in turn give the patient their unique ear shape.
